# Proprioceptive Cervicogenic Dizziness: A Narrative Review of Pathogenesis, Diagnosis, and Treatment

**DOI:** 10.3390/jcm11216293

**Published:** 2022-10-26

**Authors:** Yongchao Li, Liang Yang, Chen Dai, Baogan Peng

**Affiliations:** The Third Medical Centre of Chinese PLA General Hospital, Department of Orthopedics, 69 Yongding Road, Beijing 100039, China

**Keywords:** cervicogenic dizziness, cervical proprioception, proprioceptors, neck pain, diagnosis, management

## Abstract

Basic science and clinical evidence suggest that cervical spine disorders can lead to dizziness. The cervical spine has highly developed proprioceptive receptors, whose input information is integrated with the visual and vestibular systems in the central nervous system, acting on the neck and eye muscles to maintain the coordinative motion of the head, eyes, neck, and body through various reflex activities. When the cervical proprioceptive input changes due to the mismatch or conflict between vestibular, visual, and proprioceptive inputs, cervicogenic dizziness may occur. The diagnosis of cervicogenic dizziness can be determined based on clinical features, diagnostic tests, and the exclusion of other possible sources of dizziness. The cervical torsion test appears to be the best diagnostic method for cervicogenic dizziness. Based on the available evidence, we first developed the diagnostic criteria for cervicogenic dizziness. Treatment for cervicogenic dizziness is similar to that for neck pain, and manual therapy is most widely recommended.

## 1. Introduction

Dizziness is one of the most common reasons for consultation in adult patients [1,2]. It is an umbrella term used to describe various sensations, including vertigo, disequilibrium, lightheadedness, or presyncope (Table 1) [2]. From this perspective, vertigo is just one part of dizziness. However, in the light of the International Bárány Society for NeuroOtology [3], dizziness and vertigo are no longer subordinate but independent allelic symptoms. Dizziness and vertigo may coexist or occur sequentially (Table 2) [3]. In 1955, Ryan and Cope [4] first described dizziness caused by neck disorders as cervical vertigo, also known as cervicogenic vertigo, cervicogenic dizziness or cervical dizziness. In this review, we use cervicogenic dizziness to name this trouble. A recent clinical observation of a large number of cases (1000 cases) found that cervicogenic dizziness accounted for 89% of all dizziness, or vertigo [5]. Cervical spondylosis was one of the common causes of dizziness in the elderly in a community survey [1]. Among patients with cervical vertebral whiplash injuries, the prevalence of dizziness has been variously reported, ranging from 20% to 90% [6]. Nearly half of patients with neck pain have cervicogenic dizziness [1]. However, cervicogenic dizziness is the most controversial among all dizziness because its pathogenesis is unclear, and its diagnosis and treatment are difficult [6,7,8,9].

Cervicogenic dizziness is considered to have four different pathogenesis, but proprioceptive cervicogenic dizziness is the most common and accepted by most scholars [6]. Unlike other forms of dizziness, cervicogenic dizziness is of interest not only to neurologists but also to physiotherapists, pain physicians, and orthopedic surgeons. The purpose of this narrative review is to highlight the pathophysiology, diagnosis, and treatment of cervicogenic dizziness from the perspective of the cervical proprioceptive afferent disorder.

## 2. Pathophysiology

### 2.1. Cervical Proprioceptors and Proprioception

The sensorimotor system includes all the afferent, efferent, central integrations, and processing parts [10]. Somatosensory is the afferent component of the sensorimotor system and includes the conscious perception of pressure, temperature, vibration, pain, and proprioception [10,11]. Proprioception has often been described as our sixth sensation [12], including kinesthesia, force sensation, and joint position sense [10,11,13].

Proprioceptive information is conducted by specialized nerve endings, called proprioceptors which are situated in the joints, muscles, tendons, and skin [10,12]. Cervical proprioceptive afferents can be primarily divided into three groups: joint receptors, muscle spindle, and Golgi tendon organs (GTO) [12,14,15], which play a significant role in head-eye coordination and posture maintenance [16]. Proprioceptive information in the neck plays a crucial role in monitoring head orientation and offering a reference for the visual and vestibular receptors [2]. In physiological conditions, muscle spindles play an important role in kinesthesia GTOs conduce to the senses of heaviness and force, while cervical joint receptors may act a secondary role in proprioception [15].

Muscle spindles and GTOs react to the changes in skeletal muscle length and tension, respectively. A high density of muscle spindles has been found in the neck region of humans [16,17,18]. In general, spindle density refers to the number of spindles in wet muscle tissue per gram of muscle sample and is often used to compare the relative abundance of muscle spindles in different muscles of the same species [16,17]. In a highly cited and classic article, Kulkarni et al. [16] found that the suboccipital small muscles in human fetuses had an abundant spindle density and spindle content but lacked GTOs, making them ideal sensors for detecting the joint position and movements of craniovertebral joints. They also found that the inferior oblique muscle had a spindle density of 242/g, while the trapezius muscle had a spindle density of only 2.2/g [16]. Boyd-Clark et al. [17] found that the spindle density of longus Colli (48.6/g) was significantly higher than that of the multifidus muscle (24.3/g) in human autopsies, and the morphology, distribution, and density of spindle did not change with age. Although spindle density has been widely used in comparative studies, muscle mass has never been shown to be an appropriate reference for spindle number [18]. Banks et al. [18] revealed that spindle density itself has a nonlinear relationship with muscle mass, so direct linear comparisons of muscle spindle densities across muscle sizes are misleading. They performed an allometric analysis on the number of spindles in mammalian skeletal muscles and suggested the use of residual value as a simple way to measure the relative abundance of muscle spindle components. There was no difference in relative spindle abundance between large and small muscles as measured by residual values [18]. The longus capitis muscle, semispinalis capitis, and obliquus capitis inferior have the highest relative abundance values (7.5, 4.9, and 3.5, respectively) [18].

It is widely accepted that the joint receptors in proprioception act as joint limit detectors, playing a significant role in the sense of position near the limits of joint motion [12,15,19]. However, joint receptors are critical for the control of feedforward muscle activity and muscle stiffness via the gamma muscle spindle system [20]. Slight flexion of the upper cervical joints can lead to significant changes in the discharge rate of muscle spindle afferents in the perivertebral muscles [21]. Thunberg et al. [22] found reflex connections between receptors in the neck facet joints and fusimotoneurones of dorsal cervical muscles and the transient activation of chemically sensitive nerve endings in facet joints to be capable of triggering positive feedback loops that may produce chronic pain and stiffness in the cervical muscles. Therefore, cervical joint receptors are likely to impact postural control and head-eye movement via their influence on the muscle spindle system [20,21,22].

In 1967, Freeman and Wyke [23] classified four kinds of mechanoreceptors in the knee joints of cats. Except as free nerve endings (type IV), three types of proprioceptors are also found in human cervical facet joints [24,25] and discs [26,27], including the Ruffini corpuscles (type I), Pacinian corpuscles (type II), and GTOs (type III). However, they are much lower than the amount in the muscles. Although there are a small number of mechanoreceptor endings in the facet capsules and discs, the volume of receptors is relatively large. It is likely that receptive fields are large and that one or two mechanoreceptors may be sufficient to monitor the area of each individual facet capsule or disc [24]. Animal studies have also found that the functional proprioceptors in the facet joint capsule can be activated by low-stretching-level activities [25]. In addition, the intervertebral disc is situated on the central axis of cervical motion, and thus, proprioceptors of cervical discs are in a favorable site to monitor subtle changes in the cervical position or direction of motion. [26,28].

### 2.2. Central and Reflex Connection for Cervical Proprioceptive Signals

The neck proprioceptor can provide information on the movement and position of the head relative to the trunk but not on the movement of the head in space. However, this sensory information can affect vestibular reflexes, which function to stabilize the posture of the head, eyes, and body and to construct a sense of spatial orientation [29]. The vestibular system provides relevant information about the position of the head relative to space, while the visual system identifies the position of the head relative to the external environment [30]. By combining vestibular signals with neck proprioception information, the motion signals can be coordinately transformed into a body-centered frame of reference [31]. In addition, visual-vestibular and proprioceptive-vestibular interactions are critical for postural control and gaze [31].

**Figure 1:**
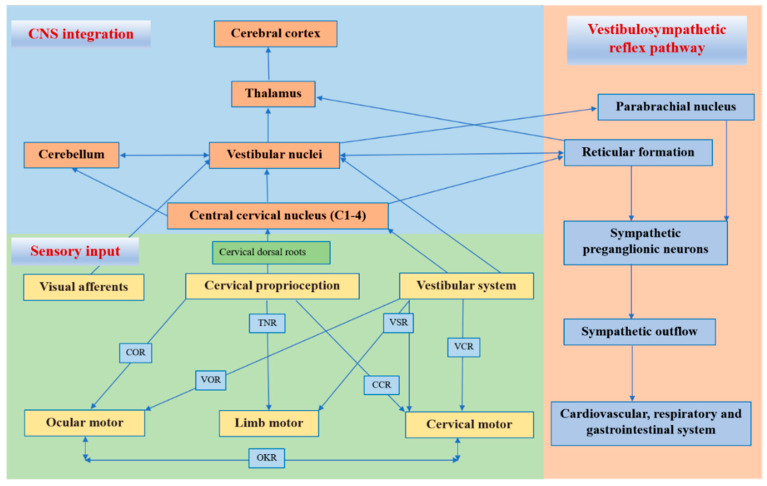
Central and reflex connection for cervical proprioceptive signals. COR = cervico–ocular reflex. VOR = vestibulocular reflex. TNR = tonic neck reflex. VSR = vestibulospinal reflex. CCR = cervico–collic reflex. VCR = vestibulo–collic reflex. OKR = optokinetic reflex.

The cervical proprioceptive system has unique central and reflex connections with the vestibular and visual systems (Figure 1). It is well recognized that the convergence of cervical proprioceptive afferents with vestibular and visual inputs at different levels of neuroaxis includes the vestibular nuclei, thalamus, and cerebral cortex [10,16,29,32,33,34]. Animal studies suggest that cervical proprioceptive afferents are transmitted to the central cervical nucleus through the dorsal root ganglion and project directly to the vestibular nucleus [35]. Neurons in the central cervical nucleus receive afferent information from the vestibular and cervical proprioceptors and provide integrated data about the head position relative to the trunk and space to the cerebellum and reticular formation [36,37]. The spinal cord can also indirectly convey input to the vestibular nuclei via the cerebellum and reticular formation [38]. Moreover, the vestibular nuclei can provide direct inputs to the reticular formation and parabrachial nucleus with projections to sympathetic preganglionic neurons of the thoracic spinal cord, which are involved in producing the rapid adjustment of circulation, digestion, and respiration necessary to maintain homeostasis (through the vestibulo-sympathetic reflex pathway) [39,40]. Reflex activities relating to the cervical, vestibular, and visual systems play a significant role in the coordination of head, neck, and eye movements (Figure 1).

### 2.3. Altered Cervical Proprioceptive Afferent and Cervicogenic Dizziness

Some studies have found that the “reversible” injury of the neck can cause defects in balance function and vision. [6]. The injection of local anesthetics in the neck is induced by nystagmus and ataxia in animals, and ataxia, a sense of falling or tilting without nystagmus, in humans [41]. In addition, the unilateral disconnection of C1–C3 dorsal roots can produce almost the same effect as a unilateral labyrinthectomy and unilateral transection of the upper cervical afferents, which can result in severe ataxia and nystagmus [42,43]. Furthermore, neck muscle vibrations in humans can induce prolonged eye position changes [44], visual illusory movements [45], and increased body sway [46].

As reviewed above, the cervical spine has very well-developed proprioceptors, and their input information integrates with the vestibular and visual systems in multiple levels of the central nervous system, which act on the neck and eye muscles through a variety of reflex activities to maintain the coordinated movement of the head, eyes, neck, and body. When cervical proprioceptive afferent activities are reduced or increased in the process of multilevel information integration in the central nervous system, cervicogenic dizziness can occur due to sensory mismatch or conflict between the vestibular, visual, and proprioceptive inputs. The conflict or mismatch of central information integration can also explain the pathogenesis of motion sickness and visual vertigo [47]. In general, abnormalities anywhere in neural pathways, from cervical proprioceptors to all levels of the central nervous system, can cause altered proprioceptive inputs and result in dizziness and instability. The function of cervical mechanoreceptors can be altered due to neck trauma, muscular spasms or fatigue, cervical degenerative disease, or neck pain [8,9].

Neck muscle spasms or fatigue can alter proprioception and postural control, and a prolonged contraction of unilateral cervical muscles can significantly increase the sensitivity of the neck proprioceptors [48]. Interstitial inflammatory mediators produced by muscle fatigue can make muscle spindle sensitive [49]. In addition, the spontaneous electromyographic activity of myofascial trigger points is consistent with the hyperactive muscle spindles [50].

Neck pain often causes proprioceptive deficits and muscle tension. Chronic pain seems to be associated with a decrease in painful muscle activity and an increase in the activity of the ipsilateral and contralateral non-painful muscles [51]. Chronic neck pain may also be associated with a reduction in the cross-sectional area of the neck muscles and deficiencies in muscle function in terms of strength, accuracy, acuity, endurance, and range of motion. [52]. However, changes in the structure and function of the neck muscles can change the discharge of proprioception, thus affecting afferent inputs and leading to changes in proprioception [53]. Cervical pain can distort sensorimotor control through side-specific changes. Even when the pain itself is significantly reduced, there can be long-term effects on proprioception [54]. In addition, neck pain can also influence the central modulation of the neck proprioceptive input and subcortical and cortical reorganization at multiple levels of the somatosensory system [55].

Cervical trauma, such as whiplash injuries, can cause cervical proprioception disorders. Neck pain, limitation of movement, and strains on the cervical muscles could modify the proprioceptive input in a whiplash-injured neck [7]. Whiplash injuries can directly damage the proprioceptive receptors in facet joints, discs, and muscles. The release of inflammatory mediators associated with trauma activates chemically sensitive nerve endings in the joints and discs [22]. Muscle morphological changes, such as fatty infiltration [56] and psychological distress when activated in a sympathetic nervous system [57], also influence the function and activity of the cervical muscles.

Cervical degenerative diseases are the most common cervical disorders in humans. Patients with cervical spondylosis are often accompanied by neck pain, neck muscle fatigue, neck stiffness, or dizziness. Most chronic idiopathic neck pain is caused by the degeneration of the cervical disc or facet joints [58]. Recent studies found that a multitude of mechanoreceptors, including Ruffini corpuscles and nociceptive receptors growing into the degenerative cervical intervertebral disc, is related to dizziness and neck pain [26,27,59]. The degenerative changes of the cervical disc are always related to inflammation and abnormal mechanical stimulation [26]. In the inflammatory setting, combined with a marked increase in the number of mechanoreceptors, it is likely that the firing characteristics of the mechanoreceptors may become overactive, which in turn induces erroneous proprioceptive afferents [26,27]. In addition, the mechanoreceptors in the cervical intervertebral discs and facet joints can control and monitor the activity of muscle spindles and paraspinal muscles [20]. The electrical stimulation of the facet joint capsule and the disc can cause the contraction of the paravertebral muscles [60]. In pathological situations, such as cervical facet osteoarthritis or intervertebral disc degeneration, the erroneous proprioceptive afferents are directly generated by the mechanoreceptors into the cervical facet joint capsules and the discs or are indirectly generated by neck pain, which becomes mismatched with the normal vestibular and visual information in multiple levels of the central nervous systems and results in the symptoms of dizziness, disorientation, and balance disturbances [26]. Cervical spinal cord or nerve root compression due to cervical myelopathy or radiculopathy can also affect proprioceptive transmission and cause proprioceptive deficits [61,62]. In addition, surgical decompression can reduce dizziness symptoms in patients with cervical root or spinal cord compression [27,63,64,65,66]. Information from the cervical muscle spindles and GTOs match each other in the appropriate cervical alignment [14,67]. Therefore, an abnormal cervical curvature in cervical degenerative diseases may cause abnormal activities in the muscle spindles and GTOs, leading to an aberrant proprioceptive input.

Cervicogenic dizziness is often accompanied by autonomic nervous disorder symptoms, such as palpitations, nausea, and vomiting. As mentioned above, neuroanatomic studies have shown that there are neural projections between the vestibular nucleus, central cervical nucleus, reticular formation, and parabrachial nucleus. Therefore, it is reasonable to believe that the sensory mismatch of vestibular, visual, and neck proprioceptive systems in the vestibular nucleus integration will affect the function of the reticular formation and parabrachial nucleus, leading to an abnormal sympathetic outflow, subsequently causing cardiovascular and gastrointestinal symptoms. In addition, sympathetic innervation is directly related to the intrafusal fibers [68], and a sympathetic outflow can intensely inhibit the proprioceptive input of cat cervical muscle spindles [21].

## 3. Diagnosis

Because of the lack of validated diagnostic tests and consistent diagnostic criteria, the diagnosis of cervicogenic dizziness is challenging and often depends on the limited diagnostic experiences of the physician.

### 3.1. Clinical Features

Dizziness from cervical disorders is a sensation of light, heavy, or full-headedness or disequilibrium accompanied by slight ataxia of stance and gait but rarely true vertigo [4,7,9,69,70]. This dizziness usually occurs in an episodic nature and can last from minutes to hours [8] and may be reproduced by a specific neck movement or position rather than a whole-body movement [4,70]. Patients with cervical dizziness often complain of neck pain, cervical stiffness, visual disturbances, nausea, vomiting, headaches, tinnitus, and palpitation. [6,7,64,66,71]. A physical examination of patients with cervicogenic dizziness can often find neck muscle tension and tenderness, zygapophyseal joint tenderness, cervical movement restrictions, hypomobile cervico-thoracic regions, and postural imbalance [69,72].

Cervicogenic dizziness is closely related to neck pain in time [6,8,9,70,73]. Neck pain is a more specific (100%) but less sensitive (68%) symptom of cervicogenic dizziness [73]. Usually, patients with neck pain accompanied by dizziness have a significantly higher disability and pain score than patients without dizziness [74]. Therefore, if the patient complains of dizziness but does not have neck pain, the diagnosis of cervicogenic dizziness can first be ruled out [6,8,9]. However, there are some exceptions in clinical practice. Some patients with severe neck pain will not experience dizziness, while others with less severe neck pain will experience dizziness. It is likely that some patients may be more sensitive to proprioception disorders or have an asymmetrical cervical input [26,72].

In clinical practice, patients with cervical spondylosis often have neck pain and dizziness. A prospective cohort study carried out by Peng et al. [65] found that there is a significant positive correlation between the reduction in dizziness intensity and the improvement of modified Japanese orthopedic association scores in patients with cervical spondylosis and dizziness after anterior cervical surgery. In addition, dizziness can also happen in patients with chronic neck pain with only cervical intervertebral disc degeneration, without a cervical spinal cord or nerve root compression on magnetic resonance imaging (MRI) [27]. The female gender, smoking, C3/4 instability, and a C3/4 Miyazaki grade ≥ IV may be the risk factors for patients with cervical spondylosis and dizziness [63]. The study also found that neck pain with dizziness was more common in women and that women with dizziness had higher levels of depression than men [74]. This may be related to the muscle spindle function, which can be affected by the emotional situation [14].

### 3.2. Diagnostic Tests

As mentioned above, patients with cervicogenic dizziness present with altered proprioception. The proprioceptive function is usually measured using two tests aimed at evaluating the sense of position or movement [10,11,75]. Theoretically, cervical joint position error (JPE) tests, which are used to measure the sense of joint position [75], and posturography, which is used to measure postural stability [76], should be potential clinical assessment tools for the diagnosis of cervicogenic dizziness. Under normal conditions, stretching the neck muscles can induce the cervico-ocular reflex (COR), which acts on the extraocular muscles to keep the eyes in a normal position [34]. When there is a lesion in the neck, the COR becomes abnormal, leading to nystagmus. Therefore, the variations in the eye movement pattern related to neck torsion might be a diagnostic marker of cervicogenic dizziness [7]. In addition, in young patients with cervicogenic dizziness without osteoarthritis, the presence of a positive Romberg test indicates that proprioception at the cervical spinal level may be impaired. However, the Romberg test is a qualitative rather than a quantitative assessment. More objective and accurate measurement tools, such as the force platform, are needed to quantitatively assess the balance function.

#### 3.2.1. Cervical Joint Position Error Test

Cervical JPE tests have been extensively applied to distinguish patients with chronic neck pain from healthy controls (Table 3) [30,75]. L’Heureux-Lebeau et al. [73] found that the specificity and sensitivity of the cervical relocalization test (a positive was defined when the average JPE was above 4.5 degrees) for the diagnosis of cervicogenic dizziness were 75% and 72%, respectively. However, the specificity and sensitivity of this test (a positive was defined when a position of JPE was greater than 4.5 degrees) were 54% and 92%, respectively. In patients with cervical spondylosis, higher pain intensity was related to greater cervical error [53]. In addition, there was a significant correlation between increasing age and an increasing error on the JPE test, with older people showing greater errors than younger people [77].

#### 3.2.2. Seated Cervical Torsion Test

The seated cervical torsion test, which requires the patient’s head to be stable and the body to rotate underneath, causes nystagmus to exceed two degrees per second, indicating a positive test (Table 3) [8,73]. The specificity and sensitivity of the seated cervical torsion test for the diagnosis of cervicogenic dizziness were 92% and 72%, respectively [73]. Moreover, cognitive variables did not affect the results of the cervical torsion tests [78]. In addition, the implementation of the seated cervical torsion test has been made easier by the recent use of video Frenzel goggles, which allow small amounts of nystagmus to be detected at the bedside [73,78]. With the advancement of technology, cervical torsion tests seem to be the best diagnostic method for diagnosing cervicogenic dizziness, but more clinical studies are still required to confirm this [78].

#### 3.2.3. Smooth Pursuit Neck Torsion Test

The smooth pursuit neck torsion test is similar to the cervical torsion test, but the operation procedure is more complicated (Table 3). Smooth pursuit neck torsion tests appear to be helpful in diagnosing cervicogenic dizziness, especially in patients with symptoms of dizziness associated with whiplash-associated disorders, as it has a specificity of 91% and sensitivity of 90% [79]. However, L’Heureux-Lebeau et al. [73] reported a specificity and sensitivity of 88% and 56%, respectively, when compared with patients with benign paroxysmal positional vertigo. In practice, smooth pursuit tests are not more sensitive and specific than cervical torsion tests [73]. However, the smooth pursuit is a complex multi-input system, which is easily affected by age, cognitive variables, and neck pain intensity [7,78]. For these reasons, the smooth pursuit test is unlikely to act as a universal role in the diagnosis of cervicogenic dizziness [7].

#### 3.2.4. Posturography

Clinically, postural sway is often measured by posturography (Table 3). Some studies suggest that dynamic posturography is an effective measurement tool for evaluating patients with suspected cervicogenic dizziness [76,80]. In addition, posturography can be a useful supplementary tool to distinguish whiplash-associated dizziness from malingerer [80]. A systematic review indicated that posturography appeared to be the only characteristic that could distinguish cervicogenic dizziness from the rest of the population [81]. In general, a normal postural sway test helps distinguish between normal people and patients with cervicogenic dizziness [7,78].

#### 3.2.5. Vestibular Laboratory Tests

Vestibular lesions are one of the usual causes of vertigo in clinical practice [7]. Because there is no specific clinical diagnostic test to determine cervicogenic dizziness, vestibular laboratory tests (such as the Dix Hallpike test, cervical vestibular-evoked myogenic potentials, and force platform) are often required to rule out dizziness and vertigo due to vestibular lesions before diagnosing cervicogenic dizziness.

#### 3.2.6. Diagnostic Blockade Test

Analgesic cervical discography [27] and the upper cervical medial branch block [82] have been reported to be valuable diagnostic tests for cervicogenic dizziness. The proprioception information from the mechanoreceptors in the cervical facet joint or intervertebral disc is transmitted to the cervical spinal cord via the nerve roots. Theoretically, if the receptor or nerve root is blocked dizziness will be reduced.

### 3.3. Imaging Features

Imaging studies can reveal structural damage or disorders in the bone or soft tissue of the neck, which increases the likelihood of diagnosing cervicogenic dizziness [7,78]. Cervical X-ray examinations can evaluate cervical curvature, instability, fracture, and degenerative changes. Computed tomography or MRI allows the survey of anatomical alterations, malformations, expansive lesions, traumatic alterations, or degenerative conditions [7]. Magnetic resonance angiography or computed tomography angiography is helpful in identifying vascular defects, which may cause dizziness in the presence of an artery compression pathology in the cervical spine.

### 3.4. Diagnostic Criteria

In the absence of a “gold standard” for the definitive diagnosis of cervicogenic dizziness, the published peer-reviewed studies supporting diagnosis and treatment rely on clusters of clinical features and diagnostic tests. Recently, a systematic review found that the most consistent diagnostic criteria stemmed from the co-occurrence of neck pain and dizziness after the exclusion of other possible causes for dizziness [83]. Based on the best evidence and reference to the diagnostic criteria of cervicogenic headaches [84] and cervicogenic somatic tinnitus [85], we propose the following diagnostic criteria for cervicogenic dizziness (Box 1).

Box 1 **Diagnostic criteria for cervicogenic dizziness.**

**Diagnostic Criteria:**
A.   Clinical, laboratory, and/or imaging evidence of a disorder or lesion within the cervical spine or soft tissues of the neck known to be able to cause dizziness.
B.   Temporal coincidence of the appearance or increase in both neck pain and dizziness.
C.   Evidence demonstrated by **at least two** of the following:
1. Dizziness has developed in temporal relation to the onset of the cervical disorder or appearance of the lesion.
2. Dizziness has significantly improved or been resolved in parallel with an improve-ment in or resolution of the cervical disorder or lesion.
3. at least two clinical diagnostic tests (cervical torsion test, cervical joint position error, or posturography) are positive.
4. Dizziness is abolished following a diagnostic blockade of a cervical structure or its nerve supply.
D.   Exclusion of other possible sources of dizziness, including the vestibular, visual, central nervous system, or psychosomatic pathologies.
**Notes:**
1.   Abnormal imaging findings of the cervical spine are common in people without dizziness; they are suggestive but do not have exact etiological evidence.
2.   Tumors, fractures, infections, and rheumatoid arthritis of the cervical spine have not been formally validated as causes of dizziness but are accepted to fulfill criteria A in individual cases.

### 3.5. Differential Diagnosis

Cervicogenic dizziness is an exclusion diagnosis. Therefore, it is significant to rule out other possible sources of dizziness before establishing the diagnosis of cervicogenic dizziness. The most common differential diagnoses of cervicogenic dizziness are benign paroxysmal positional vertigo, Ménière’s disease, vestibular migraines, neuritis, and so on. Clinical features in the differential diagnosis of cervicogenic dizziness with their diagnostic criteria are presented in the previously published reviews on this topic [7,69].

## 4. Treatment

Cervicogenic dizziness should be treated in the same manner as neck pain. The majority of patients can be effectively improved by strict conservative treatment, but a small number of refractory dizziness patients, who have failed to respond to various non-surgical treatments, can be treated by surgery. Most published articles on the treatment of dizziness are of poor quality and are based on different diagnostic criteria. So far, some high-quality randomized controlled trials have been published.

### 4.1. Conservative Treatment

Based on clinical practice and the available literature, the non-surgical treatment of cervicogenic dizziness mainly includes pharmacological treatment, physical therapy, vestibular rehabilitation, and acupuncture.

#### 4.1.1. Pharmacological Treatment

Clinical drugs used to treat cervicogenic dizziness include non-steroidal anti-inflammatory drugs (NSAIDs), antidepressants, muscle relaxants, and Chinese herbal medicines [6,7,8,86]. NSAIDs have the effects of analgesia, anti-inflammatory, and the inhibition of abnormal proprioception from the muscle spindles [6]. Antidepressants may improve emotional symptoms, reduce sympathetic outflow, and inhibit the proprioceptive input from muscle spindle afferents in the neck muscles [14]. Muscle relaxants such as iperisone can effectively relieve pain and stiffness in patients with cervical spondylosis. Chinese herbal medicines have been extensively applied in cervicogenic dizziness because of their empirical efficacy in suppressing pain and improving blood circulation. Recently, a systematic review and meta-analysis found that, when combined with other treatments, Chinese herbal medicines may improve the treatment effect of cervicogenic dizziness without serious adverse reactions [86].

#### 4.1.2. Physical Therapy

Physical therapy is absorbed in reducing neck pain, muscle stiffness and spasms, so as to improve activity and ability. It is a reasonable treatment for those patients who are considered to have cervicogenic dizziness accompanied by either cervical whiplash or degenerative disorders [72,74,78,87]. Bittar et al. [88] found that Helical patches provide a continuous heat flow, resulting in tense muscle relaxation, which seems to be a valid treatment for cervicogenic dizziness. In a prospective study of the case series, Minguez-Zuazo and colleagues [89] found that the therapeutic education and exercise of patients with cervicogenic dizziness seemed to reduce neck disability and dizziness. Recently, Moustafa et al. [87] found that the use of extension traction devices to restore cervical lordosis and reduce forehead posture had a positive effect on patients with cervicogenic dizziness during long-term follow-up. Therefore, appropriate physiotherapy rehabilitation for cervicogenic dizziness should include the rehabilitation of the cervical structure, which may lead to greater and more lasting functional improvements [87].

Proprioception is mostly acquired and trainable [13]. Manual therapy can restore the normal movement of facet joints, reduce pain, and decrease muscle hypertonicity, thereby normalizing cervical proprioceptive and biomechanical functions [90]. Up to now, manual therapy has been the most widely studied method for cervicogenic dizziness. Several published prospective randomized controlled studies [91,92,93,94,95,96,97,98,99,100] and systematic reviews [90,101,102] provide evidence to support the use of manual therapy for the treatment of cervicogenic dizziness. Theoretically, a well-integrated vestibulo-cerebellar system can compensate for the change in neck proprioception afferents in the case of cervicogenic dizziness. Therefore, we can conclude that vestibular rehabilitation can enhance the vestibulo-cerebellar system to recover adaptive ability when the normal cervical proprioceptive afferent is impaired [101]. Yacovino and Hain^7^ thought that manual and vestibular physiotherapy seems to be the most reasonable treatment strategy for cervicogenic dizziness.

#### 4.1.3. Acupuncture and Pharmacopuncture

A systematic review by Hou et al. [103] based on the low or very low quality of evidence suggests that acupuncture appears to be a promising treatment for cervicogenic dizziness. In another review included in the randomized controlled trials, Kim and Cho found that pharmacopuncture could improve the efficacy of routine treatment for cervicogenic dizziness [104].

### 4.2. Surgical Treatment

In a cross-sectional study, Reddy et al. [53] found that patients with cervical spondylosis had impaired proprioception compared with healthy subjects. According to the existing basic and clinical findings, Liu et al. [105] summarized that the degeneration of the cervical intervertebral disc can be considered a significant cause of dizziness. It has been found that anterior cervical surgery or percutaneous disc decompression is an effective treatment for cervical spondylosis with dizziness. Four consecutive case series studies have found that percutaneous disc decompression and nucleoplasty are effective, minimally invasive, and low-complication procedures for cervicogenic dizziness [64,71,106,107,108]. Li et al. [64] showed that percutaneous cervical nucleoplasty for cervical degenerative diseases with both neck pain and cervicogenic dizziness has been satisfactory in clinical outcomes in the short- and mid-term follow-up and fair in the long-term follow-up of 6-years. Therefore, percutaneous cervical decompression and nucleoplasty can be used as complementary procedures to make up the gap between conservative treatments and open surgery [108]. Numerous studies [63,65,66], including a multicenter prospective cohort study [65], have indicated that anterior cervical surgery can effectively improve dizziness in patients with cervical radiculopathy and/or myelopathy. In addition, anterior cervical surgery can also improve neck pain and dizziness in patients with only cervical intervertebral disc degeneration but not with a cervical spinal cord or nerve root compression based upon an intradiscal block test [27].

## 5. Conclusions

Any cervical disorder or lesion may cause dizziness. The most convincing pathogenesis of cervicogenic dizziness is a sensory mismatch between the abnormal neck proprioceptive input because of trauma, muscular spasms, or degenerative diseases and the input from the vestibule and visual system in the process of multilevel information integration in the central nervous system. The diagnosis of proprioceptive cervicogenic dizziness can be determined based on clinical features, diagnostic tests, and the exclusion of other possible sources of dizziness. The treatment of proprioceptive cervicogenic dizziness is similar to that of neck pain, and manual therapy is most widely recommended.

## Figures and Tables

**Table 1:** Main categories of dizziness.

Category	Description
Vertigo	A sense of spinning experienced even when someone is perfectly still.
Disequilibrium	A loss or lack of equilibrium or stability.
Presyncope	Feeling of losing consciousness or blacking out.
Lightheadedness	Feeling a little woozy or faint.

**Table 2:** Definition of dizziness and vertigo by the International Bárány Society for NeuroOtology.

Classification	Description
Dizziness	The sensation of disturbed or impaired spatial orientation without a hallucinatory or distorted sense of motion.
Vertigo	The sensation of self-motion when no self-motion is occurring or the sensation of distorted self-motion during an otherwise normal head movement.

**Table 3:** Diagnostic tests for cervicogenic dizziness.

Test	Mechanism	Purpose	Measurement (Unit)	Method	Positive Test
Cervical joint position error	Improper cervicocollic reflex inhibition	Measure joint position sense	Neutral head position or target error (degree or centimeter)	Participants sit in chairs and face the target on a wall 90 cm away. They are blindfolded there and a specific laser pointer is placed on top of their heads. Participants are asked to move their head away from the target when the laser pointer is right in the center of the target. After returning to the center, the error is evaluated between the starting position and the final position.	The joint position error in one position is above 4.5 degrees
Seated cervical torsion test	Impaired cervicoocular reflex	Measure cervicoocular reflex	Ratio of eye to target motion in neutral and torsion positions	The participants are seated on a stool or chair and eye movements are recorded when ocular fixation is inhibited. Participants turn trunk 90 degrees to right, keeping their heads still. Then, return to the center, turn the trunk 90 degrees to the left, and return to the center. Hold each position for 30 s, and the observer stabilizes the head in all positions.	Nystagmus > 2 degrees persecond at any of the four positions
Smooth pursuit neck torsion test	Impaired cervicoocular reflex	Measure cervicoocular reflex	Ratio of eye to target motion in neutral and torsion positions	Participants focus their eyes on a moving target and keep their head still, in a neutral position, and rotate their torso relative to the head (torsion). Record the speed of eye movement while tracking the target in the vertical or horizontal plane. The average gain is calculated as a parameter defining smooth pursuits. The difference between the average gain of the neutral position of the head and left and right torsion of the head on the body is calculated to determine the difference.	Nystagmus > 2 degrees persecond in left or right neck torsion (excluding a spontaneous nystagmus)
Posturography	Quantitative test of the vestibulospinal reflex.	Measure postural control stability in upright stance in either static or dynamic conditions	Sway area (cm^2^) or total sway path (mm)	Records were taken while standing on a power platform with both eyes open and closed.	Disturbed postural control

## Data Availability

Not applicable.
